# Evaluating the Effectiveness of Reference Solvent Solubility Calculations for Binary Mixtures Based on Pure Solvent Solubility: The Case of Phenolic Acids

**DOI:** 10.3390/molecules30224444

**Published:** 2025-11-18

**Authors:** Piotr Cysewski, Tomasz Jeliński, Rafal Rozalski, Fabian Lesniewski, Maciej Przybyłek

**Affiliations:** 1 Department of Physical Chemistry, Faculty of Pharmacy, Collegium Medicum in Bydgoszcz, Nicolaus Copernicus University in Toruń, Kurpińskiego 5, 85-950 Bydgoszcz, Poland; tomasz.jelinski@cm.umk.pl; 2 Department of Clinical Biochemistry, Faculty of Pharmacy, Collegium Medicum in Bydgoszcz, Nicolaus Copernicus University in Toruń, Karłowicza 24, 85-950 Bydgoszcz, Poland; rafalr@cm.umk.pl (R.R.); fabian.lesniewski@cm.umk.pl (F.L.)

**Keywords:** solubility, binary solvent, reference solvent, COSMO-RS, machine learning, nuSVR, phenolic acids, caffeic acid, ferulic acid, intermolecular interactions

## Abstract

Predicting the solubility of active pharmaceutical ingredients (APIs) in binary solvent mixtures is a major challenge in formulation science, as physics-based models often fail to capture complex, non-additive mixing effects. This study presents a robust machine learning (ML) framework to overcome this limitation, enabling accurate predictions from pure solvent data alone and molecular descriptors derived from COSMO-RS (computed with COSMOtherm). Firstly, our experimental knowledge of binary solvent mixtures solubility was expanded through newly measured data of caffeic and ferulic acids in aqueous mixtures of DMF, DMSO, and 4-formylmorpholine (4-FM). These new data, combined with values in the literature, formed a comprehensive dataset of 1636 points for ten phenolic and benzoic acids. To build a predictive model, a systematic methodology was developed, with the acronym of DOO-IT (Dual-Objective Optimization with ITerative features pruning), which automates descriptor selection and hyperparameter optimization to yield a maximally parsimonious and generalizable model. An exhaustive, multi-run stability analysis identified a final 10-descriptor nuSVR model as the optimal solution. This model demonstrated outstanding predictive power, achieving an R^2^ of 0.988 and MAE equal to 0.0514 on a held-out test set, vastly outperforming standard COSMO-RS approaches. Interpretation of the selected descriptors revealed that the model successfully learns to correct for non-ideal mixing by integrating a baseline solubility reference with specific solute–solvent and solvent–solvent interaction terms. This work delivers both a practical tool for reducing experimental screening and a powerful, transferable methodology for developing robust QSPR models for complex chemical systems.

## 1. Introduction

Phenolic acids (PhAs) constitute a structurally diverse and biologically relevant subclass of plant-derived polyphenols, typically categorized into hydroxybenzoic and hydroxycinnamic acids. They are widely distributed in fruits, vegetables, grains, and beverages such as coffee and tea, and are frequently encountered in both free and conjugated forms [1,2,3]. Interestingly, beyond their well-known plant-based sources, phenolic acids have also been identified in edible mushrooms such as *Boletus badius*, *Cantharellus cibarius*, and *Pleurotus ostreatus*, which contain significant quantities of protocatechuic, ferulic, and sinapic acids, indicating that fungi may represent an underestimated dietary reservoir of these compounds [4]. Notably, PhAs, along with other naturally occurring phenolic compounds, exhibit a broad spectrum of bioactivities, including antioxidant effects, as demonstrated by their capacity to scavenge reactive oxygen species and protect cellular components from oxidative damage [5,6], and anti-inflammatory effects, implemented via the inhibition of COX-2 and pro-inflammatory cytokines [7,8]. They also demonstrate antimicrobial activities, particularly through phenolic-rich extracts that inhibit bacterial growth [9], and cytoprotective actions, as shown in studies using phenolic-acid-loaded nanocarriers to protect human mononucleated cells from oxidative injury [10]. Since many of these effects depend on physicochemical behavior in solution, solubility has become a key focus in understanding and optimizing their functional potential.

The solubility behavior of phenolic acids in various solvent systems is primarily governed by their combination of polar functional groups and aromatic character, leading to consistent trends across structurally diverse representatives such as salicylic, caffeic, ferulic, and sinapic acids. These compounds generally exhibit low solubility in water but significantly improved solubility in polar organic solvents such as methanol, ethanol, acetone, and acetonitrile [11,12]. Methanol has been widely reported as the most effective solvent for extracting hydroxybenzoic and hydroxycinnamic acids from various sources, including *Pistacia atlantica*, *Juglans regia*, and *Tamarindus indica*, yielding higher levels of chlorogenic, ferulic, caffeic, and protocatechuic acids compared to ethanol [13,14,15]. On the other hand, some studies have shown that aprotic polar solvents, such as 1,4-dioxane and tetrahydrofuran, may demonstrate even greater solubilizing power than alcohols, as evidenced by the case of salicylic acid [16].

One common strategy to enhance solubility is the use of mixed solvent systems. Numerous studies have demonstrated that binary solvents provide significantly higher solubility and extracting properties for various phenolic compounds compared to pure solvents, due to the synergistic effects of solvent polarity and hydrogen bonding [17,18,19]. However, synergy is not always observed, and in some cases, binary mixtures may fail to enhance solubility as expected [20,21]. This underscores the complex nature of solubility phenomena for phenolic acids in binary systems, where both molecular structure and solvent–solute interactions play critical roles. To address these challenges, a variety of computational approaches have been developed to enable a deeper understanding and accurate prediction of solubility behavior across diverse solvent environments.

Various computational methods have been developed to predict the solubility of phenolic acids and structurally related compounds, addressing the limitations of purely experimental approaches. Thermodynamic models such as UNIFAC, UNIQUAC-SAC, and NRTL-SAC have been widely used for solubility prediction in both pure and binary solvents, offering semi-predictive capabilities based on molecular group contributions and solvation parameters [22,23,24]. Among the most prominent and broadly applicable tools is the Conductor-like Screening Model for Real Solvents (COSMO-RS), which integrates quantum chemical surface polarization calculations with statistical thermodynamics to predict solubility, hydrogen bonding, and activity coefficients in diverse solvent systems [25,26].

Recent studies have increasingly combined quantum chemical models with machine learning (ML) to enhance solubility prediction. COSMO-RS-derived descriptors have been integrated with neural networks and gradient boosting algorithms to accurately predict solubility in ionic liquids (ILs), and deep eutectic solvents (DESs), often outperforming standalone models [27,28,29]. These hybrid approaches enable solvent screening, model interpretability, and robust prediction across diverse chemical spaces. For drug-like compounds, COSMO-RS descriptors have been paired with artificial neural networks to estimate solubility, gradient-boosted tree models have also been developed for this purpose, and Gaussian process regression has been used as a post-processing correction to COSMO-SAC predictions [30,31,32,33]. For gases in ILs and DES, σ-profile features from COSMO-RS have been combined with neural networks for CO_2_ in chemically reactive DES [27], with XGBoost for CO_2_ and N_2_ in ILs [29], and with mechanism-informed linear models that encode thermodynamic structure for fluorinated and related systems [28]; complementary IL design pipelines integrate random forest property models with COSMO-RS screening [34], and equation-of-state analyses provide a mechanistic baseline for gas solubility in fluorinated ILs [35]. As such, the synergy of quantum calculations with ML holds significant promise for optimizing extraction systems and guiding rational formulation design.

The aim of this study is to develop a predictive model capable of estimating the solubility of phenolic acids in binary solvent systems based on their solubility in pure solvents. The model will be constructed using COSMO-RS-derived molecular descriptors combined with machine learning techniques, allowing for the capture of non-linear solvent–solute interactions. To support and validate the modeling approach, experimental solubility data will be determined for two representative phenolic acids, caffeic acid (CAF) and ferulic acid (FER), in selected aqueous–organic mixtures containing efficient polar aprotic solvents.

## 2. Results and Discussion

### 2.1. Solubility Measurements of Caffeic and Ferulic Acids

**Figure 1:**
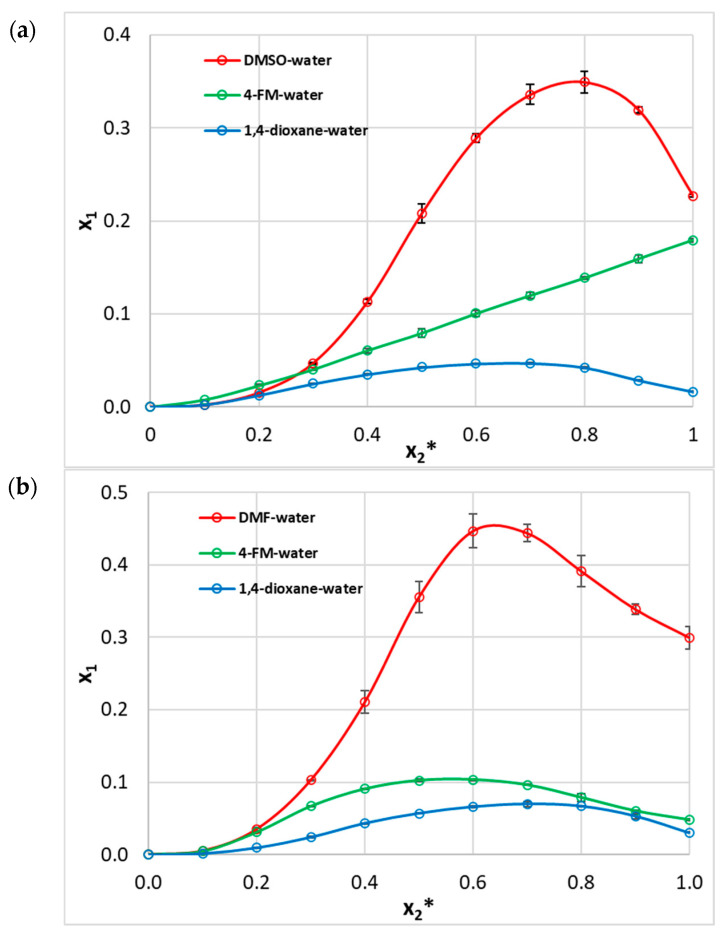
Mole fraction solubility of caffeic acid (CAF) and ferulic acid (FER) at 25 °C in binary aqueous–organic solvent systems: (**a**) CAF and (**b**) FER. Solubility is expressed as the mole fraction of solute (x_1_) and plotted against the mole fraction of the organic component (x_2_*) in the solute-free mixture.

As part of the experimental section of this study, the solubility of two representative phenolic acids (namely caffeic and ferulic acids) was determined in mixtures comprising aprotic organic solvents and water, which acts as a protic solvent. In the available literature, solubility data for caffeic acid, ferulic acid, and their analogs are predominantly reported for alcoholic media, whereas aprotic solvents have been explored to a lesser extent [11,36,37]. The results presented herein enrich and extend the existing dataset with new solubility values for systems that had not been previously investigated. The obtained experimental results are presented in Figure 1, with the corresponding tabulated solubility data provided in Table S1 (Supplementary Materials). For caffeic acid (CAF), the solubility profiles differ considerably across the tested systems. In the 1,4-dioxane–water system, solubility increases with x_2_*, from 9.0 × 10^−5^ at x_2_* = 0.0 (pure water) to a maximum of 4.698 × 10^−2^ at x_2_* = 0.7, followed by a gradual decline to 1.634 × 10^−2^ in pure 1,4-dioxane (x_2_* = 1.0). A much more pronounced enhancement is observed in the DMSO–water system, where solubility reaches a maximum of 34.931 × 10^−2^ at x_2_* = 0.8 and subsequently decreases to 22.675 × 10^−2^ in pure DMSO. Notably, this system yields the highest solubility observed for CAF among all solvent systems investigated. In the 4-formylmorpholine (4-FM) aqueous mixtures, solubility increases monotonically across the entire composition range, reaching 17.932 × 10^−2^ in pure 4-FM. No distinct maximum is observed in this case, indicating continuous improvement with increasing organic content.

For ferulic acid (FER), similar non-linear solubility trends are observed. In the 1,4-dioxane–water system, solubility increases markedly from 5.0 × 10^−5^ at x_2_* = 0.0 (pure water) to 6.979 × 10^−2^ at x_2_* = 0.7, followed by a decrease to 3.025 × 10^−2^ in pure 1,4-dioxane (x_2_* = 1.0). In the 4-FM–water system, the maximum value of 10.342 × 10^−2^ occurs at x_2_* = 0.6, followed by a decline to 4.814 × 10^−2^ at x_2_* = 1.0. The DMF–water system yields the highest solubility values observed for FER across all systems: 44.661 × 10^−2^ at x_2_* = 0.6 and 44.380 × 10^−2^ at x_2_* = 0.7. This then drops to 29.943 × 10^−2^ in pure DMF. The marked difference between the peak and the value in the neat solvent emphasizes the contribution of water at moderate concentrations in promoting solubility.

### 2.2. Identifying an Optimal Predictive Model via the DOO-IT Framework

**Figure 2:**
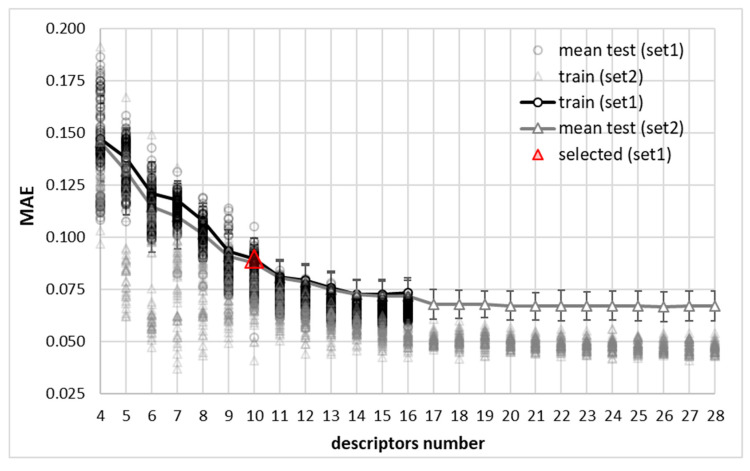
Model stability across descriptor complexity. The mean absolute error (MAE) is plotted for the best models selected from the Pareto front over 150 independent DOO-IT runs (75 per descriptor set). Training performance (dots) and mean test performance with standard deviation (solid line and shaded area) are shown. The red triangle indicates the final 10-descriptor champion model, selected for its superior stability and predictive performance. The selected molecular features were the following: log(x_API_^COSMO^) (1.29), E_vdW,API_ (0.88), E_misfit,solvent_ (0.51), E_misfit,API_ (0.44), E_int,API_ (0.40), ΔE_HB_ (0.36), E_HB,solvent_ (0.32), μ_API_ (0.31), E_HB,API_ (0.30), μ_solvent_ (0.19); and the final parameters are the following: {‘nu’: 0.296612135954871, ‘C’: 48.50288872197616, ‘log10_gamma_scale’: 0.9977796098840708}.

The central challenge in predicting the solubility of a solute in a binary solvent mixture is to effectively capture the complex, non-additive interactions between the three components. To address this, the predictive model was developed using the systematic DOO-IT (Dual-Objective Optimization with ITerative feature pruning) methodology [38,39], which is designed to identify the most accurate and parsimonious model from a given set of descriptors. To ensure the robustness of our findings and to thoroughly map the solution space, the entire DOO-IT procedure was repeated fifteen independent times. The results of this exhaustive analysis are summarized in Figure 2, which plots the results of the multi-criteria selection scheme applied for the final model selection. The figure reveals two critical insights into the nature of this prediction problem.

Firstly, the optimization landscape is exceptionally sensitive and complex. There is a wide variance in MAE of both train and test scores for any given number of features what is clearly indicated by the scatter gray symbols denoting runs of two descriptors sets. This demonstrates that a simplistic approach with a single-run modeling approach is highly susceptible to finding suboptimal solutions. This highlights the strong, non-linear interdependencies between the descriptors, where the predictive power of one feature is highly contingent on the presence of others. Many feature combinations, even with optimized hyperparameters of the nuSVR regressor, result in poor models, underscoring the difficulty of the task. Secondly, and most importantly, despite this challenging landscape, the rigorous data-driven multi-criteria framework is able to select solutions that balance fit quality, parsimony and persistence. Hence, the single best model was objectively selected from the family of the cast number of candidates. It is interesting to note that 10-descriptor model was selected irrespective of the initial number of descriptors (16 for set1 and 28 for set 2). This reveals a well-defined and surprisingly narrow “basin of excellence”, where the globally optimal models are exclusively located. This is a highly significant finding: it suggests that the complex behavior of solubility in binary mixtures can be captured by a relatively small, core set of physical descriptors, provided that this exact set is identified. Figure 2 demonstrates the methodology’s power to uncover a parsimonious and highly optimal model from a challenging, non-convex search space.

### 2.3. Model of Phenolic Acids Solubility in Binary Mixtures

**Figure 3:**
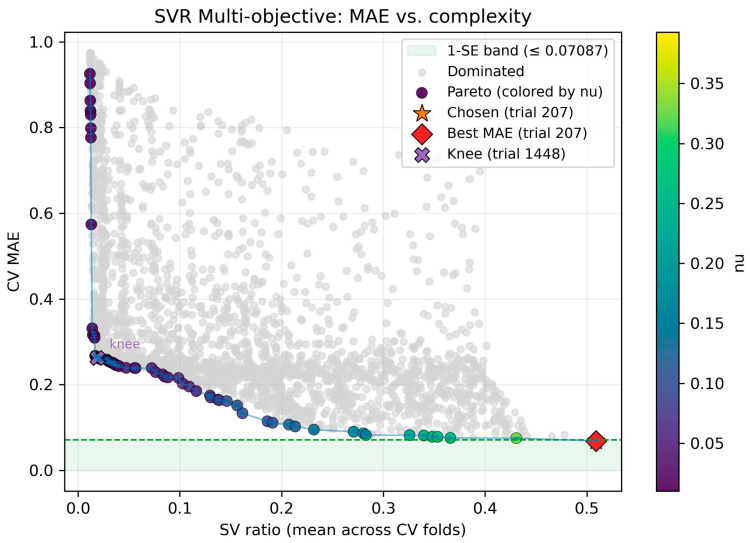
Dual-objective optimization for model selection. The plot illustrates the trade-off between model accuracy, measured by cross-validated mean absolute error (CV MAE), and complexity, measured by the support vector ratio (SV ratio), for all evaluated nuSVR models with the optimal 10-descriptor set. The Pareto front (dark purple points), representing non-dominated solutions, is highlighted. The final model (trial 207, orange star) was selected by applying the one-standard-error (1-SE) rule, which identifies the simplest model within a statistically equivalent performance band (green shaded region). In this case, the selected model coincided with the single most accurate candidate on the Pareto front.

The results of the dual-objective optimization for the 10-descriptor feature set, from which the final model was selected, are presented in Figure 3. Each point in the plot represents a unique nuSVR model, positioned according to its cross-validated mean absolute error (CV MAE) and its complexity, as measured by the mean support vector (SV) ratio. The plot clearly illustrates the fundamental trade-off between these two competing objectives. The set of non-dominated solutions forms a distinct Pareto front (dark purple points), which traces the best achievable accuracy for any given level of model complexity. Models to the right of the front, in the “dominated” region (gray points), are objectively inferior, as a more accurate and simpler model exists. The color of the points along the front corresponds to the nu hyperparameter, revealing a clear trend: simpler models with a low SV ratio are achieved with lower nu values, while more complex models with a high SV ratio correspond to higher nu values.

The final model was selected by applying the statistically robust one-standard-error (1-SE) rule. This involved first identifying the model with the absolute lowest cross-validation mean absolute error (MAE) on the Pareto front (trial 207, red diamond). The standard error of this model’s performance was then used to establish a 1-SE threshold (MAE ≤ 0.07371), defining a region of statistically equivalent performance (green shaded region). The model from trial 207 was the sole candidate within this performance band and, as it also possessed the lowest complexity, it was selected as the final champion. While alternative heuristics like identifying the “knee” of the curve (e.g., trial 1448) exist, the 1-SE rule provides a more conservative and principled methodology that explicitly favors parsimony, thereby maximizing the likelihood of robust generalization to unseen data.

### 2.4. The Challenge of Predicting Binary Mixture Solubility

**Figure 4:**
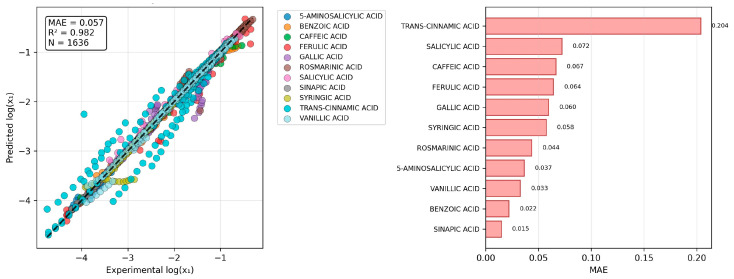
Predictive performance of the final model. The parity plot compares experimental and predicted solubility (log(x_1_)) for all 1656 data points, colored by solute. The model exhibits high accuracy (MAE = 0.057) and explanatory power (R^2^ = 0.982). The solute-wise distribution of points shows no systematic bias for the majority of compounds illustrating consistent performance across diverse phenolic acid structures. The distribution of the MAE for each solute is provided in the right panel.

The central challenge in predicting the solubility of a solute in a binary solvent mixture lies in accurately capturing the complex, non-additive interactions between the three components. Physics-based models, like COSMO-RS, provide a powerful starting point, but they can exhibit significant systematic errors. Hence, relying only on COSMOtherm outcomes is not recommended unless only a qualitative characteristic is sufficient. The challenge for reference solvent computations is especially visible when the experimental solubilities in both neat solvents are provided as reference points for extreme ranges of binary composition, including pure solvent state. Such referencing of solubility is particularly unreliable when there is a large discrepancy in the solute’s solubility between the two neat solvents (e.g., for systems containing water and DMSO). In these cases, the model struggles to interpolate between the two reference points, often leading to large prediction errors. This finding is highly significant: it demonstrates that simply providing experimental data from the endpoints (the neat solvents) is insufficient in capturing the complex, synergistic, or antagonistic mixing effects that govern solubility in the binary mixture. There is a clear need for a more sophisticated method that can learn these non-linear correction factors. This is precisely the problem the DOO-IT machine learning framework was designed to solve. The final 10-descriptor model selected via the DOO-IT framework’s rigorous multi-criteria scheme demonstrates predictive performance and generalization for the domain of selected set of phenolic acids. The parity plot (Figure 4) visually confirms the model’s accuracy, with the vast majority of the 1656 data points clustering tightly around the ideal line of unity. The overall high coefficient of determination (R^2^ = 0.982) and low mean absolute error (MAE = 0.057) on the entire dataset attest to the model’s ability to capture the complex, non-linear relationships between the COSMO-RS-derived molecular descriptors and solubility in binary solvent mixtures. The points are colored by solute, revealing a relatively uniform distribution of errors across different molecules, which indicates that the model does not exhibit a strong, systematic bias towards any particular chemical subclass within the dataset. The results, summarized in the accompanying table, reveal consistently outstanding performance for most compounds, with MAE values below 0.06 and R^2^ values exceeding 0.99 for ten out of the eleven solutes. A notable exception is trans-cinnamic acid, which exhibited a higher MAE (0.151) and a lower R^2^ (0.935). This outlier behavior warrants further investigation but may be attributed to its distinct molecular structure, which features an extended, unconjugated carbon chain that is not present in the other, more heavily hydroxylated and substituted benzoic acid derivatives. This structural uniqueness could lead to specific solute–solvent interactions that are not as comprehensively captured by the selected descriptor set, highlighting a potential boundary of the model’s applicability domain.

As documented in Figure 2, the descriptor set used by the final model reveals that accurate solubility prediction in binary mixtures requires integrating three fundamental aspects of molecular interactions. First, the model establishes a baseline solubility reference through the COSMO-RS predicted solubility (importance: 1.29), which serves as the dominant feature. Interestingly, despite the relatively poor predictive potential of this descriptor alone, the model does not discard this quantum–chemically derived estimate but rather uses it as a foundation for refinement. The second set represents the intrinsic solute properties. The model incorporates key characteristics of the solute (API) that define its interaction potential. This includes its capacity for van der Waals interactions (E_vdW,API_, 0.88), its hydrogen bonding ability (E_HB,API_, 0.30), its polarity (μ_API_, 0.31), and its internal energy state (E_int,API_, 0.40; E_misfit,API_, 0.44). These features describe the API’s inherent “personality” in a solvation environment. The third contribution comes from explicit solvent-mediated interactions. Crucially, the model includes descriptors that explicitly account for the non-ideal, multi-component nature of the binary solvent system. This is the core mechanism by which it corrects the pure solvent baseline. The hydrogen bonding energy difference (ΔE_HB_, 0.36) and the solvent dipole moment (μ_solvent_, 0.19) are critical for capturing the cooperative or competitive effects between the two solvent components. These descriptors allow the model to learn how the solvent mixture’s own internal networking, such as water–organic solvent H-bonding, either enhances or diminishes the solvation power for the specific API. The high importance of specific interaction terms like E_misfit,solvent_ S (0.51) and ΔE_HB_ (0.36) demonstrates that the model successfully learns to correct for non-ideal mixing effects that pure solvent predictions cannot capture. This descriptor combination effectively creates a molecular-level picture where the API’s intrinsic solubility is modulated by its specific interactions with both solvent components and the competitive interactions between the solvents themselves.

### 2.5. The Spectrum of Model Generalization

A central challenge in the development of predictive Quantitative Structure–Property Relationship (QSPR) models is to rigorously assess their true generalization capabilities. The choice of validation strategy is not merely a procedural step but fundamentally defines the claims one can make about a model’s utility for new, unseen data. This validation challenge lies in assessing generalization capability across chemical space and it manifests differently depending on the validation strategy employed and the extent of chemical diversity within the dataset. Below, a proposal to rephrase the problem in terms of traditional dichotomy between interpolation and extrapolation approaches is presented.

Random cross-validation, the predominant validation approach in QSPR studies, primarily evaluates interpolation performance—the model’s ability to predict properties for compounds structurally similar to those in the training set. Validation via random splits, where data points are randomly assigned to training and test sets, primarily assesses a model’s interpolative power. In this regime, the chemical space of the test set is, by statistical design, highly similar to that of the training set. It is highly probable that for any given compound in the test set, a close structural analog exists in the training data. Therefore, a model that performs well under this scheme is robust and reliable at making predictions within the well-characterized boundaries of its known chemical domain [40]. This is a crucial test of the model’s ability to learn the fine-grained relationships between descriptors and the target property, but it does not guarantee performance on entirely novel chemical scaffolds. In contrast, LOCO cross-validation and Extrapolative Generalization, where all data associated with a single solute are reserved for testing, represents a far more stringent assessment of a model’s extrapolative capabilities [41]. This procedure simulates a real-world scenario where the model is tasked with predicting the property for a compound that is structurally distinct from anything it has been trained on. Consequently, LOCO tests the model’s ability to generalize its learned “physicochemical rules” to new regions of chemical space. This is intrinsically linked to the concept of the model’s applicability domain (AD), which defines the chemical space where its predictions are considered reliable [42]. A significant performance degradation in LOCO compared to random splits is therefore not an indication of a flawed model, but rather a quantitative measure of the challenge of extrapolation.

The distinction between generalization modes becomes particularly pronounced in datasets with limited chemical diversity, as is common in mixture property prediction where experimental constraints often restrict the number of unique solutes investigated. The performance differential between random splits and LOCO validation is intrinsically linked to the density of chemical space coverage [43]. In sparsely populated chemical spaces—typical of specialized solubility studies with only several unique solutes—each compound may occupy a distinct region with minimal structural overlap with others. Under these conditions, LOCO validation represents extreme extrapolation, often resulting in significantly degraded performance metrics compared to random validation. As datasets expand to encompass greater chemical diversity, the validation landscape undergoes a fundamental transformation. With increasing compound coverage, the chemical space between training examples becomes more densely populated, effectively transforming what was once extrapolation into interpolation between known chemical regions [44]. This phenomenon can be understood through the lens of applicability domains: as more compounds are added, the domain becomes more continuous, with fewer true “gaps” requiring extrapolation [45]. This has immense implications for model development with limited data. From the perspective of solubility modeling, especially in specialized applications such as ionic liquid solubility or deep eutectic solvent design, the data shortage is the norm rather than the exception. This prohibits the adoption of an extrapolation perspective, and the split framework provides crucial insights for model development and validation. Hence, in sparse chemical spaces, the stability of selected descriptors across multiple random splits becomes a critical indicator of model robustness. Features that consistently emerge as important across diverse data partitions are more likely to capture fundamental physicochemical relationships rather than dataset-specific artifacts. Furthermore, the advantages of chemistry-informed feature engineering become non-trivial. Indeed, when chemical diversity is limited, incorporating domain knowledge through physics-based descriptors can partially compensate for sparse coverage by encoding fundamental molecular interactions. Then, the progressive validation strategies employing systematic datasets grow, allowing one to monitor the convergence rates between random and LOCO validation; this then provides insights into chemical space coverage adequacy. Rapid convergence suggests good domain representation, while persistent gaps indicate the need for targeted compound selection to fill chemical space voids.

The dual-validation approach adopted in this paper is particularly valuable given our dataset’s characteristics. While LOCO provides a conservative estimate of performance on truly novel solutes, the random-split framework was essential for ensuring the fundamental quality of the model itself. By leveraging repeated random splits within our multi-objective optimization and stability selection workflow, one can focus on identifying a subset of molecular descriptors that are consistently predictive across different data partitions. This computationally intensive procedure, facilitated by our DOO-IT workflow, is designed to favor descriptors that encode fundamental physicochemical principles over those that are artifacts of a specific training set composition [38,46]. This focus on descriptor stability ensures that we are not merely overfitting to the known compounds but instead are also building a model architecture that captures more generalizable relationships. Such a model is both more robust for interpolation and better poised to benefit from future dataset expansion. As we strategically add new, diverse solutes to our dataset, the “holes” in our chemical space will shrink. Consequently, the LOCO task will become less extremely extrapolative, and we anticipate the performance of the LOCO and random-split frameworks will begin to converge, as both increasingly measure true generalization within our expanding domain of interest [47].

While stringent extrapolative validation approaches, like Leave-One-Compound-Out (LOCO), are essential for gauging a model’s performance on entirely novel chemical classes, it is crucial to recognize the significant industrial and scientific value of models validated for high interpolative accuracy. These models, typically assessed via random-split cross-validation, excel in scenarios where the goal is to optimize, refine, or explore a well-defined chemical or compositional space. In these contexts, precision within a known domain is paramount, and such models serve as powerful tools for accelerating discovery and reducing experimental costs. One can point out several key domains where these “interpolative” models find direct, real-world application. As the first domain for applications of this research, one can suggest that leading optimization might be achieved in Medicinal Chemistry. Here, the drug-discovery pipeline is a multi-stage process. After a “hit” compound with the desired biological activities is identified, the project enters the lead optimization phase. The goal here is not to find a completely new chemical scaffold, but to synthesize and test dozens or hundreds of close structural analogs of the initial hit to systematically improve properties like potency, selectivity, solubility (aqueous and non-aqueous), metabolic stability, and permeability, while reducing toxicity. Hence, a QSAR model can be built using an initial set of 30–50 synthesized analogs; this, validated with random splits, establishes a robust relationship between small structural modifications and the resulting property changes. Then, this can be used to perform a virtual screening of thousands of proposed but unsynthesized analogs. The model accurately predicts which modifications are most likely to yield improvements, allowing the team to prioritize the synthesis of only the top 5–10% most promising candidates. This dramatically reduces the number of compounds that need to be made and tested, saving months of effort and significant resources. In this context, a LOCO-validated model would be less relevant, as the goal is to intentionally work within a single, highly constrained chemical series.

Another potential domain that might benefit from the interpolation framework is product refinement and adaptation in formulation science. Industries such as cosmetics, paints, agrochemicals, and food science rely on complex multi-component mixtures. The performance of these formulations, including, among others, shelf-life, viscosity, active ingredient stability, and color fastness, are sensitive functions of the precise composition. The obtained model predicts key properties like drying time and stability as functions of component ratios. It can then be used to rapidly screen alternative, compliant solvents and predict the minor adjustments in other component percentages needed to maintain or improve product performance. The model is interpolating within the known space of “acceptable formulation ingredients,” a task for which random-split validation is perfectly suited.

In addition, one can also suggest the reaction optimization in process chemistry as a potential area of interpolative QSPR model applications. Optimizing the conditions of a chemical synthesis to maximize yield and purity is a classic application of Design of Experiments (DoE). Machine learning models are the modern engine for analyzing DoE data. Here, the “chemical space” is the parameter space of the reaction. The model’s interpolative power allows the company to run a minimal set of experiments while still confidently identifying the best process conditions, increasing efficiency and reducing waste.

It is not hard to enumerate many more practical domains, where the key factors for the random-split model deployment offer real leverage in the following ways:

(i) Defined application domains with clear boundaries for the chemical space, where predictions are valuable;
(ii) Representative training data covering the operational chemical space;
(iii) Incorporating regular model updates while new data are available to maintain chemical space coverage;
(iv) Clear uncertainty quantification by implementing applicability domain checks to flag extrapolation attempts;
(v) Ensuring that the users of model applications understand the models’ limitations and appropriate use cases.

## 3. Materials and Methods

### 3.1. Materials

Caffeic acid (≥98.0%, CAS: 331-39-5) and trans-ferulic acid (≥99.0%, CAS: 537-98-4) were purchased from Merck Polska (Warsaw, Poland). The organic solvents used for solubility measurements and in analytical procedures included methanol (analytical grade, CAS: 67-56-1, Chempur, Piekary Śląskie, Poland), 1,4-dioxane (99.8%, CAS: 123-91-1, Chempur), dimethyl sulfoxide (DMSO, ≥99.9%, CAS: 67-68-5, Merck Polska, Warsaw, Poland), 4-formylmorpholine (99%, CAS: 4394-85-8, Merck), and dimethylformamide (DMF, ≥99.0%, CAS: 68-12-2, Merck Polska, Warsaw, Poland). Acetic acid (85%, CAS: 64-19-7) was obtained from Chempur (Piekary Śląskie, Poland) and used as a modifier in the analytical procedure.

### 3.2. Solubility Determination Procedure

First, an excess amount of either caffeic acid or ferulic acid was placed in a test tube, which was subsequently filled with 10 mL of the selected solvent to obtain saturated solutions. In the case of binary mixtures, the organic solvent was mixed with water in appropriate molar fractions. Water was also used as a single-component solvent. For each system, three replicate samples were prepared.

The prepared samples were placed in an Orbital Shaker ES-20/60 incubator from Biosan (Riga, Latvia) and shaken for 24 h at 25 °C. The temperature was maintained with an accuracy of ±0.1 °C. During the equilibration process, the samples were agitated at a rate of 60 rpm while being thermostated.

Following equilibration, the samples were filtered through PTFE syringe filters with a 0.22 µm pore size. To prevent precipitation during this step, all test tubes, syringes, and filters were preheated to the corresponding temperature of the solution. Subsequently, an aliquot of the filtrate was transferred to a vial for chromatographic analysis.

Quantification of caffeic acid was carried out using a high-performance liquid chromatography (HPLC) system consisting of two 515 pumps, a 2667 sample manager, and a 2996 photodiode array detector (Waters, Milford, CT, USA). Prior to injection, samples were diluted with methanol and injected into a Luna C18 column (250 × 10 mm) equipped with a guard column, both from Phenomenex (Torrance, CA, USA). The injection volume was 100 µL, and the column temperature was maintained at 30 °C. The mobile phase consisted of 0.5% acetic acid (A) and acetonitrile (B), applied with a linear gradient at a flow rate of 5 mL/min: initially 2% B; 1–7 min, 2–50% B; 7–9 min, 50% B; 9–9.5 min, 50–2% B; and 9–18 min, 2% B. The UV absorbance was monitored in the range of 210–400 nm. The chromatographic system was operated with MassLynx 4.1 software (Waters, Milford, CT, USA). Quantitative analysis was performed using the TargetLynx application at 293 nm. All samples were analyzed in triplicate (technical replicates).

Ferulic acid was quantified using ultra-performance liquid chromatography with UV detection (UPLC–UV). The system included the Acquity Binary Solvent Manager, Acquity Sample Manager, and Acquity Column Manager (Waters, Milford, CT, USA). Prior to analysis, samples were diluted with methanol, and 0.3 µL was injected. Separation was performed on a ACQUITY UPLC CSH C18 column (1.7 µm, 2.1 × 30 mm, Waters, Milford, CT, USA) maintained at 40 °C. UV detection was carried out at 293 nm using a tunable dual-wavelength UV/Vis detector (TUV, Waters, Milford, CT, USA). The linear gradient employed 10 mM ammonium acetate (A) and acetonitrile (B) as mobile phases: 0–0.2 min, 5% B; 0.2–1 min, 5–50% B; 1–1.2 min, 50% B; 1.2–1.25 min, 50–5% B; and 1.25–2.5 min, 5% B. The system was controlled with MassLynx 4.2 software (Waters, Milford, CT, USA), and quantitative analysis was performed using the TargetLynx application. Each sample was analyzed in three technical replicates.

To calculate mole fractions of the solutes, the density of each saturated solution was determined by weighing 1 mL of the sample using an analytical balance (RADWAG AS 110.R2 PLUS; Radwag, Radom, Poland).

### 3.3. COSMO-RS Computations

Application of the COSMO-RS framework [48,49,50,51,52] requires appropriate representation of molecular diversity. This is achieved by performing a conformational analysis prior to the determination of any thermodynamic properties. For this purpose, the default protocol was applied, taking advantage of the COSMOconf (version 2023, BIOVIA COSMOlogic)/TURBOMOLE (version 7.7, 2023, TURBOMOLE GmbH) tandem for the generation of the most representative structures for all solutes and solvent molecules. The applied protocol is consistent with previously published schemes [53,54,55]. For each molecule, up to ten low-energy conformations were determined for both gas and condensed phases, the latter accounting for solvent effects under the conductor-like screening model. The resulting “cosmo” and “energy” files were generated using the BP_TZVPD_FINE_24.ctd parameter set, essential for thermodynamic calculations in COSMOtherm, which requires application of the RI-BP/TZVP//TZVPD-FINE level of theory.

### 3.4. Reference Solvent Computations

In this study, COSMOtherm was not run with fusion data (T_m_, ΔH_fus_, ΔG_fus_). Using fusion data for solid solutes is known to be challenging for the present acids, the reference-solvent option was adopted and the calculations were anchored to experimental solubilities in the neat solvents [56,57], COSMO-RS allows for declaring the values for reference solvent solubility in the input files, which is used to determine the values of Gibbs free energy and used in the iterative procedure of chemical potential computations in the bulk saturated phase. Unfortunately, this is not accurate enough and cannot be used for quantitative purposes. However, the values of solubility estimated in such a way can be used for further refinement using a machine learning approach. Hence, the collection of phenolic acid solubility in neat solvents and binary solvent mixtures and different temperatures was collected from the literature for augmenting measurements conducted specifically for the purpose of this study. In the case of multiple reports of solubility in neat solvents, the averaged values were used for reference solubility computations. It is also worth adding that the published data are occasionally incomplete or inconsistent, and data curation is indispensable. Hence, the data collected from the literature were carefully inspected and accordingly grouped into four categories. Category #1 comprised data that were complete and coherent and were included in the dataset as provided by authors in original publications without any curation. The data were regarded as complete and consistent if the measurements for pure solvents were conducted at the same temperatures as for different compositions and all mixtures had the same ratio of solvents. Category #2 is a collection comprising such cases for which inconsistencies were noticed between the temperatures of solubility measurements in pure solvents and binary mixtures, but the same compositions were preserved for all ratios of solvents. In such a case, the data were complete but not usable in the direct way for reference solvent computations, as it is necessary to have solubility in mixtures and neat solvents at the same temperatures. Although there is an option in COSMOtherm enabling the declaration of both reference solubility value and temperature, this introduces additional uncertainty, and the danger of incompatibility between set 1 and set 2 might arise. Hence, such systems were interpreted in terms of Buchowski-Ksiazczak (λh-model) [58,59] or Van’t Hoff three-parameter models (VH3). The error introduced by fitting is marginal, and back-computed solubility data were included in the dataset. It is worth mentioning that the latter model does not require the values of melting temperature for fitting purposes but needs at least four data points for fitting. The λh-model is a two-parameter equation and hence can be applied to systems with as few as three measurements. Category #3 comprises cases with inconsistency in concentration but consistency in the temperatures of measurements. This enabled the utilization of the Jouyban–Acree [60,61,62,63,64] three-parameter model for back-computing and standardization of data. Finally, there were incomplete cases, for example, lacking pure solvent solubility, as it was not provided by authors. However, using averaged values reported by other authors enabled the inclusion of these data in the dataset for model development. Category #4 includes such systems for which incoherencies in the concentration of binary mixtures were noticed. In such cases, the prepared binary mixtures differed, depending on the temperature and required standardization by fitting to theoretical models. This approach was used to smooth the data for each binary composition and to estimate solubility at the same temperature and at the same ratio of pure solvents used for preparing the binary mixtures. This study focuses exclusively on category #1 systems; the full list is available in the Supplementary Dataset (RefSol_data.xlsx). The dataset includes results obtained here alongside data compiled from research reports [65,66,67,68,69,70,71,72,73,74,75,76,77,78,79,80,81].

### 3.5. Molecular Descriptors

The generation of predictive models requires a quantitative representation of molecular structures through descriptors. For this work, a set of descriptors was selected based on several key principles: they must be calculable from chemical structure alone, incorporate relevant physical conditions such as temperature and composition, and have a clear physical basis to aid in model interpretation. These criteria are essential for enabling high-throughput screening of novel compounds and for ensuring the model’s applicability across different experimental conditions. In this study, the molecular descriptors were derived from COSMO-RS theory, forming two primary sets for model development. The first set was composed of interaction energies obtained from solubility calculations.

From the COSMO-RS output, five core descriptors were extracted for each solute: the total intermolecular interaction energy (E_int,API_), its components—electrostatic misfit (E_misfit,API_), hydrogen bonding (E_HB,API_), and van der Waals contributions (E_vdW,API_)—and the chemical potential (μ_API_). Corresponding solvent descriptors (E_int,solvent_, E_misfit,solvent_, E_HB,solvent_, E_vdW,solvent_, μ_solvent_) were computed as the mole-fraction-weighted average of the individual solvent components in the solute-free mixture. The descriptor set was expanded by including the relative difference between each solute and solvent descriptor. The COSMO-RS-predicted solubility, log(x_API_^COSMO^), was also incorporated. This constituted set 1 holding 16 descriptors.

The second set of descriptors (set 2) comprised descriptors of set 1 and an additional twelve, derived based on σ-potential distributions. The standard COSMO-RS output, comprising 61 data points across a charge density range of −0.03 to +0.03 e/Å^2^, was condensed by averaging values over 0.005 e/Å^2^ intervals. This produced a 12-step function capturing three key regions of the σ-profile: the hydrogen bond donor (HBD1–4, −0.03 to −0.01 e/Å^2^), hydrophobicity (HH1–4, −0.01 to +0.01 e/Å^2^), and hydrogen bond acceptor regions (HBA1–4, +0.01 to +0.03 e/Å^2^). Four descriptors were defined for each region, yielding a total of 24 σ-potential descriptors for the solute, the solvent, and their relative difference.

These two descriptor sets served as independent sources of chemical information for modeling the solubility of eleven organic acids. Descriptor definitions and units are summarized in Table S2a, and the complete per-point values are included in RefSol_data.xlsx (Supplementary Materials). The compounds studied included ten phenolic acids—caffeic acid, ferulic acid, gallic acid, rosmarinic acid, salicylic acid, sinapic acid, syringic acid, trans-cinnamic acid, 5-aminosalicylic acid, and vanillic acid—as well as benzoic acid. The complete dataset, comprising 1636 data points, is provided in the Supplementary Materials. It includes all experimental and computed solubility values, molecular descriptors, and set classifications necessary to reproduce this work.

### 3.6. Machine Learning Protocol

#### 3.6.1. Core Algorithm and Data Preprocessing

The machine learning workflow was centered on the nu–Support Vector Regression (nuSVR) algorithm [82], chosen for its demonstrated ability to effectively model the complex, non-linear relationships often present in QSPR studies [83,84,85]. To handle these non-linearities, the Radial Basis Function (RBF) kernel was selected. The RBF kernel is a powerful and flexible choice, capable of mapping features into an infinite-dimensional space, which allows it to model intricate decision boundaries while requiring the tuning of only a single parameter, gamma. The optimization of the nuSVR hyperparameters was conducted as follows: the regularization parameter C and the nu parameter were directly optimized. The kernel coefficient gamma, which dictates the influence of each support vector, was optimized via a guided, data-driven strategy. For each optimization cycle, a baseline gamma_base value was heuristically determined from the median pairwise squared Euclidean distance of the training data subset [86,87]. The optimizer then refined this anchor by searching for an optimal logarithmic scaling factor. This approach focuses the search on a physically relevant scale, enhancing optimization efficiency.

A critical aspect of our modeling strategy was the use of non-deterministic, repeated data splitting to ensure robust model evaluation. For each of the 150 independent DOO-IT runs, the full dataset (N = 1636) was randomly partitioned into a training set (80%) and a test set (20%). This approach prevented the model’s performance from being dependent on a single, arbitrary data split and allowed for a stability analysis of the selected features and parameters. All molecular descriptors in the training set were standardized by removing the mean and scaling to unit variance using the StandardScaler from scikit-learn [88,89]. As SVR algorithms are sensitive to feature scaling, this step ensures that no single descriptor disproportionately influences the model due to its magnitude. The same scaling transformation was subsequently applied to the test set.

#### 3.6.2. Dual-Objective Optimization Protocol

To explicitly manage the inherent trade-off between model accuracy and simplicity, a dual-objective optimization (DOO) strategy was implemented using the Optuna framework (v. 3.2) [90,91,92]. The TPE sampler within Optuna was configured to simultaneously minimize two competing objectives, which were evaluated using a 5-fold cross-validation scheme on the training data. The first objective was predictive accuracy, quantified by the mean absolute error (MAE). The second objective was model complexity, quantified by the mean support vector (SV) ratio. The SV ratio is calculated for each fold as the number of support vectors divided by the number of training samples in that fold, providing an intrinsic measure of complexity for nuSVR models. A model with a lower SV ratio is considered more parsimonious.

Hence, the outcome of a dual-objective optimization is a set of solutions forming a Pareto front. This front consists exclusively of non-dominated solutions. A solution is considered non-dominated if no other solution exists that is superior in one objective without being inferior in the other. In other words, to improve a non-dominated solution with respect to one objective, a trade-off in the form of a degradation in the other objective must be accepted. Conversely, a dominated solution is one for which at least one other solution exists that offers better performance in one objective while being no worse in the other, making it an objectively suboptimal choice.

#### 3.6.3. Iterative Model Refinement and Candidate Selection

The framework employs an iterative backward pruning methodology to integrate feature selection directly into the optimization process. This automatic procedure relies, therefore, on both dual-objective optimization and iterative features pruning (DOO-IT). The procedure begins with the complete descriptor set. A full DOO is executed, producing a Pareto front of non-dominated models. From this front, a single candidate model for the current iteration is selected, governed by the 1-Standard Error (1-SE) rule [93,94]. This involves identifying the most accurate model on the front and defining a performance threshold based on its standard error; the simplest model (lowest SV ratio) within this threshold is then chosen. Once a candidate is selected, its features are ranked based on permutation importance with 10 repeats [95]. The least impactful descriptor is then eliminated, and the procedure repeats with a new, full DOO on the reduced feature set. This cycle continues until a specified minimum number of features is reached, generating a series of robust, parsimonious candidate models at each level of complexity.

#### 3.6.4. A Data-Driven, Multi-Criteria Framework for Model Selection Based on Stability and Performance

Selecting a final, robust model from the family of candidates generated by the iterative DOO-IT procedure required a rigorous, data-driven framework that balances predictive performance with model parsimony and chemical interpretability. Unlike our previous work [38], which relied on the corrected Akaike Information Criterion (AICc) [96,97], we replaced this theoretically ambiguous criterion for nuSVR models with a practical multi-tiered strategy [39]. This strategy was applied to the 150 candidate models (from 75 independent 80/20 splits for each of two descriptor pools) generated by the DOO-IT procedure. First, architectural optimization identified the optimal descriptor count through stability analysis. We selected models that consistently appeared across multiple runs while maintaining a test set MAE within one standard error of the global minimum. This approach, inspired by the one-standard-error rule, was enhanced with an empirical stability threshold (≥30% frequency per descriptor count) to ensure the selection of a parsimonious and reproducible model architecture. Subsequently, for final model deployment, a specific model instance was chosen from the architecturally optimal group using a composite scoring system. This score balanced predictive accuracy (50% weight), explanatory power (30% weight via R^2^), and generalization capability (20% weight via the train-test performance gap). The selected model also demonstrated high descriptor stability, prioritizing molecular features that consistently appeared across independent runs.

This dual emphasis on both model architecture and specific feature set ensures that the deployed model not only exhibits strong predictive performance but is also built upon a chemically meaningful and reproducible descriptor combination. The final model was validated through comprehensive residual analysis, applicability domain assessment, and external validation where available, providing a transparent, empirically grounded foundation for practical solubility prediction.

The DOO-IT framework was implemented as a fully automated pipeline using Python 3.10 [98] with the scikit-learn [99], Optuna [92], and pandas [100] libraries. To rigorously assess solution stability, the entire procedure was repeated fifteen independent times. Each dual-objective optimization within this process was configured to run for 2000 trials, ensuring a comprehensive exploration of the solution space.

## 4. Conclusions

In this work, we addressed the significant challenge of accurately predicting API solubility in binary solvent mixtures, a task where traditional physics-based models like COSMO-RS can exhibit substantial systematic errors. We have successfully developed and rigorously validated a parsimonious, 10-descriptor nuSVR model that demonstrates outstanding predictive power, achieving an R^2^ of 0.988 and MAE equal to 0.0514 on a held-out test set. This data-driven model provides a practical and high-fidelity tool for predicting binary mixture solubility using only data derived from the neat solvent components, thereby offering a direct path to significant reduction in the experimental effort required for solvent screening and formulation design.

The success of this model is underpinned by our systematic DOO-IT methodology, which navigates the complex, non-convex optimization landscape to identify a simple and robust solution. By interpreting the final descriptor set, we revealed that the model’s success lies in its ability to learn a sophisticated, non-linear correction factor that accounts for non-ideal mixing effects. It achieves this by synergistically combining a baseline solubility reference with specific descriptors that characterize both the energetic “personality” of the solute and the crucial solvent–solvent interactions within the binary mixture.

Ultimately, this study makes two key contributions. First, it delivers a specific, validated, and practical tool for an important challenge in pharmaceutical science. Second, it provides a clear demonstration of how a carefully designed machine learning framework can overcome the inherent limitations of physics-based approaches, learning the subtle, non-additive phenomena that govern complex solution chemistry. This work serves as a compelling blueprint for developing hybrid and data-driven models to accelerate materials discovery in other complex chemical systems. Crucially, our multi-run stability analysis confirmed that while the optimization landscape is highly non-convex, a systematic search can consistently isolate a simple, physically meaningful model—proving that complex mixture phenomena can be captured with parsimony.

## Data Availability

The original contributions presented in this study are included in the article/Supplementary Material. Further inquiries can be directed to the corresponding author.

## Supplementary Materials

The following supporting information can be downloaded at: https://www.mdpi.com/article/10.3390/molecules30224444/s1, Supplementary Materials: Table S1: Experimental results; Table S2a: Dataset description; Table S2b: Reference of the source of the solubility data; RefSol_data.xlsx.
