# Supplementary material for: Evaluating the Effectiveness of Reference Solvent Solubility Calculations for Binary Mixtures Based on Pure Solvent Solubility: The Case of Phenolic Acids
Source: Molecules. 2025 Nov 18;30(22):4444. doi: 10.3390/molecules30224444 (PMC12655719; doi:10.3390/molecules30224444)
Supplement: Supplementary file 1 [file molecules-30-04444-s001.zip › molecules-3913318-supplementary_revised.pdf]

Supplementary Materials

Evaluating the effectiveness of reference solvent solubility calculations of binary mixtures based on pure solvent solubility: the case of phenolic acids

Piotr Cysewski [1,*], Tomasz Jeliński [1], Rafal Rozalski [2], Fabian Lesniewski [2] and Maciej Przybyłek [1,*]

[1]Department of Physical Chemistry, Faculty of Pharmacy, Collegium Medicum in Bydgoszcz, Nicolaus Copernicus University in Toruń, Kurpińskiego 5, 85-950 Bydgoszcz, Poland;

[2]Department of Clinical Biochemistry, Faculty of Pharmacy, Collegium Medicum in Bydgoszcz, Nicolaus Copernicus University in Toruń, Karłowicza 24, 85-950 Bydgoszcz, Poland;

Table of Content

S1. Experimental results 2

Table S1. Experimental solubility data for caffeic acid (CAF) and ferulic acid (FER) in binary aqueous–organic solvent systems at 25 °C. Solubility is expressed as the mole fraction of solute (x_1); x_2* denotes the mole fraction of the organic solvent in the solute-free binary mixture. SD refers to the standard deviation of replicate measurements... 2

S2. Dataset description 4

Table S2a. Description of the data used for machine learning. 4

Table S2b. Reference of the source of the solubility data. 6

S1. Experimental results

Table S1. Experimental solubility data for caffeic acid (CAF) and ferulic acid (FER) in binary aqueous–organic solvent systems at 25 °C. Solubility is expressed as the mole fraction of solute (x_1); x_2* denotes the mole fraction of the organic solvent in the solute-free binary mixture. SD refers to the standard deviation of replicate measurements.

x_2^*	$x_1 \times 10^2$	SD × 10^2
**CAF in 1,4-dioxane-water**		
0.0	0.009	0.001
0.1	0.243	0.002
0.2	1.269	0.033
0.3	2.495	0.014
0.4	3.504	0.055
0.5	4.260	0.071
0.6	4.647	0.075
0.7	4.698	0.093
0.8	4.232	0.076
0.9	2.850	0.034
1.0	1.634	0.020
**CAF in DMSO-water**		
0.1	0.209	0.002
0.2	1.527	0.028
0.3	4.718	0.095
0.4	11.316	0.211
0.5	20.824	1.025
0.6	28.915	0.476
0.7	33.598	1.081
0.8	34.931	1.158
0.9	31.904	0.316
1.0	22.675	0.146
**CAF in 4-FM-water**		
0.1	0.785	0.022
0.2	2.306	0.097
0.3	4.043	0.153
0.4	6.072	0.233
0.5	7.935	0.498
0.6	10.036	0.282
0.7	11.977	0.302
0.8	13.855	0.100
0.9	15.930	0.438
1.0	17.932	0.164
**FER in 1,4-dioxane-water**		
0.0	0.005	0.000
0.1	0.182	0.006
0.2	0.977	0.052
0.3	2.424	0.105

x_2*	$x_1 \times 10^2$	SD × 10^2
0.4	4.337	0.036
0.5	5.703	0.085
0.6	6.599	0.113
0.7	6.979	0.345
0.8	6.688	0.049
0.9	5.327	0.347
1.0	3.025	0.070
**FER in DMF-water**		
0.1	0.509	0.022
0.2	3.521	0.253
0.3	10.301	0.064
0.4	21.073	1.527
0.5	35.541	2.143
0.6	44.661	2.327
0.7	44.380	1.221
0.8	39.127	2.127
0.9	33.868	0.734
1.0	29.943	1.525
**FER in 4-FM-water**		
0.1	0.455	0.002
0.2	3.087	0.089
0.3	6.667	0.042
0.4	9.057	0.108
0.5	10.235	0.214
0.6	10.342	0.109
0.7	9.620	0.055
0.8	7.888	0.544
0.9	6.043	0.031
1.0	4.814	0.095

S2. Dataset description

The whole dataset used for machine learning purposes was collected in MS Excel file named RefSol_data.xlsx, which provides all necessary data for reproduction. In particular, the sheet named "data" collects experimental solubility data along with all descriptors. The notation is explained in the Table S2.b.

Table S2a. Description of the data used for machine learning.

Quantity	Description
log(x_exp)	Experimental solubility collected as decadal logarithm of mole fraction
log(x_solub)	COSMO-RS solubility obtained from reference-solvent (anchored) runs; decadal logarithm of mole fraction; used as a descriptor/baseline, not as a target.
ΔGfus[kJ/mol]	Values the Gibbs free energies computed for every solute based on the fusion data: ΔG_{fus}=ΔH_{fus}-TΔS_{fus} $\Delta C_{p,fus}$≈ΔS_{fus}≈$\Delta H_{fus}/T_m$
**SET 1**	
dmu	Relative value of chemical potentials (μ): $$dmu = mu_{API} - mu_{DES}$$
dE_tot	Relative value of the total interaction energies (denoted in the output of COSMO-RS computations as "Total mean interaction energy in the mix (H_int)"): $$dE\_tot = E_{API}^{tot} - E_{DES}^{tot}$$
dE_Misfit	Relative value of the electrostatic contribution to intermolecular interaction energies (denoted in the output of COSMO-RS computations as "Misfit interaction energy in the mix (H_MF)"): $$dE\text{Misfit} = E_{API}^{\text{Misfit}} - E_{DES}^{\text{Misfit}}$$
dE_HB	Relative value of the hydrogen bonding contribution to intermolecular interaction energies (denoted in the output of COSMO-RS computations as "H-Bond interaction energy in the mix (H_HB)": $$dE\text{\_HB} = E_{API}^{\text{HB}} - E_{DES}^{\text{HB}}$$
dE_vdW	Relative value of the non-bonding contribution to intermolecular interaction energies (denoted in the output of COSMO-RS computations as "VdW interaction energy in the mix (H_vdW)": $$dE\text{\_HB} = E_{API}^{\text{vdW}} - E_{DES}^{\text{vdW}}$$
mu1_sat	Chemical potential of the solute in the bulk saturated phase of the neat reference solvent obtained from anchored COSMO-RS runs: $$mu1\_sat = mu_{API}$$
E1_tot_sat	The values of the total interaction energies of solute $$E1\_tot\_sat = E_{API}^{tot}$$
E1_Misfit_sat	Value of the electrostatic contribution to intermolecular interaction energies the solutes: $$E1\text{\_Misfit\_sat} = E_{API}^{\text{Misfit}}$$
E1_HB_sat	Values of the hydrogen bonding contribution to intermolecular interaction energies of the solutes $$E1\text{\_HB\_sat} = E_{API}^{\text{HB}}$$
E1_vdW_sat	value of the non-bonding contribution to intermolecular interaction energies of the solutes $$E1\text{\_HB\_sat} = E_{API}^{\text{vdW}}$$
mu_solvent	Chemical potential of the solute-free binary solvent mixture evaluated at the data-point composition x_2 (x_1 = 0). Calculated as a mole-fraction-weighted sum of component chemical potentials: $$mu\_solvent = mu_{DES} = \sum_{i=1}^{N=2} x_i^* \cdot mu_i \quad .$$

	computed as a weighted sum of components contributions, where x_i^* represents the mole fraction of i-th component in solute free solution.
E_tot_solvent	Values of the total interaction energies of solvent $$E_{int,solvent} = E_{solvent}^{int} = \sum_{i=1}^{N=2} x_i^* \cdot E_i^{tot} \cdot$$
E_Misfit_solvent	Values of the electrostatic interaction energies of solvent $$E_{misfit,solvent} = E_{solvent}^{Misfit} = \sum_{i=1}^{N=2} x_i^* \cdot E_i^{Misfit} \cdot$$
E_HB_solvent	Values of the hydrogen bonding interaction energies of solvent $$E_{HB,solvent} = E_{DES}^{HB} = \sum_{i=1}^{N=2} x_i^* \cdot E_i^{HB} \cdot$$
E_vdW_solvent	Values of non-bonding interaction energies of solvent $$E_{vdW,solvent} = E_{DES}^{vdW} = \sum_{i=1}^{N=2} x_i^* \cdot E_i^{vdW} \cdot$$
**SET 2** (SET 1 augmented with the relative σ-potential contributions expressed as difference between solute and solvent values)	
d_HBD1	hydrogen bond donor in the range (-0.030,0.025 e/Å²)
d_HBD2	hydrogen bond donor in the range (-0.025, -0.020 e/Å²)
d_HBD3	hydrogen bond donor in the range (-0.020, -0.015 e/Å²)
d_HBD4	hydrogen bond donor in the range (-0.015, -0.010 e/Å²)
d_HH1	hydrophobicity in the range (-0.010, -0.005 e/Å²)
d_HH2	hydrophobicity in the range (-0.005, 0.000 e/Å²)
d_HH3	hydrophobicity in the range (0.000, +0.005 e/Å²)
d_HH4	hydrophobicity in the range (+0.005, +0.010 e/Å²)
d_HBA1	hydrogen bond acceptor in the range (+0.010, +0.015 e/Å²)
d_HBA2	hydrogen bond acceptor in the range (+0.015, +0.020 e/Å²)
d_HBA3	hydrogen bond acceptor in the range (+0.020, +0.025 e/Å²)
d_HBA4	hydrogen bond acceptor in the range (+0.025, +0.030 e/Å²)

Table S2b. Reference of the source of the solubility data.

code	year	first author	DOI
1	2025	this work	this work
2	2016	Aydi	10.1021/acs.jced.6b00008
3	2016	Zibetti	10.1016/j.molliq.2016.01.061
4	2016	Zibetti	10.1016/j.molliq.2016.01.061
5	2016	Aydi	10.1021/acs.jced.6b00008
6	2017	Noubigh	10.1021/acs.jced.7b00333
7	2016	Dali	10.1016/j.molliq.2016.07.063
8	2016	Dali	10.1016/j.molliq.2016.07.063
9	2012	Noubigh	10.1016/j.jct.2012.06.022
10	2013	Noubigh	10.1016/j.molliq.2013.07.015
11	2016	Dali	10.1016/j.molliq.2016.07.063
12	2016	Noubigh	10.1016/j.molliq.2016.04.095
13	2019	Noubigh	10.1016/j.molliq.2018.09.131
14	2019	Noubigh	10.1016/j.molliq.2018.09.131
15	2017	Haq	10.1111/jphp.12786
16	2020	Shakeel	10.1007/s10973-020-09451-y
17	2017	Shakeel	10.1016/j.molliq.2016.11.009
18	2009	Matsuda	10.1021/je800475d
19	2009	Matsuda	10.1021/je800475d
20	2009	Matsuda	10.1021/je800475d
21	2009	Matsuda	10.1021/je800475d
22	2016	Zhang	10.1021/acs.jced.5b00619
23	2022	Shakeel	10.1016/j.molliq.2021.118057
24	2018	Sandeepa	10.1021/acs.jced.8b00025
25	2018	Sandeepa	10.1021/acs.jced.8b00025
26	2018	Sandeepa	10.1021/acs.jced.8b00025
27	2018	Sandeepa	10.1021/acs.jced.8b00025
28	2018	Sandeepa	10.1021/acs.jced.8b00025
29	1916	Marden	10.1021/ja02263a014
30	2020	Moradi	10.1016/j.molliq.2020.112774
